# Implementing a Climate Health Education Curriculum for Emergency Medicine Trainees and Faculty

**DOI:** 10.5811/westjem.50824

**Published:** 2026-05-03

**Authors:** Eric Lewis, Courtney M. Smalley, Matthew Kostura

**Affiliations:** * The MetroHealth System, Cleveland, Ohio; † Cleveland Clinic Lerner College of Medicine of Case Western Reserve University, Cleveland Clinic Health System, Department of Emergency Medicine, Cleveland, Ohio

## Abstract

As climate-related health impacts intensify, emergency physicians (EP) increasingly encounter patients whose conditions are influenced by environmental change. To provide care for climate- vulnerable patients in the emergency department (ED), EPs should be educated on the impacts of climate change. The goal of our intervention was to provide a structured climate health educational curriculum to attending physicians, residents, and medical students and assess the perceived effectiveness of the curriculum. A longitudinal climate health curriculum was delivered in a four-part lecture series over the course of three months to medical students, postgraduate year 1–3 emergency medicine residents, and academic emergency attending physicians. We measured learners’ perceived knowledge pre- and post-curriculum with surveys assessing four core areas: 1) climate change topics; 2) climate impacts on human health; 3) confidence in treating medical conditions exacerbated by climate change; and 4) climate change solutions. At the completion of the three-month curriculum, the learners reported a statistically significant improvement in perceived level of knowledge in overall climate health topics in 87.5% (28/32 concepts with *P* < .05) of concepts assessed during the climate health education curriculum. Specifically, learners reported a perceived knowledge improvement in concepts of general climate change (6/6 topics, *P* < .05), impacts on human health (7/8 topics, *P* < .05), confidence in treating medical conditions exacerbated by climate change (8/9 topics, *P* < .05), and knowledge of climate solutions (7/9 topics, *P* < .05). Overall, learners reported a higher median likelihood of implementing individual climate solutions after the conclusion of the climate health education curriculum, although this was not statistically significant (5.0 vs 7.0, *P* = .073). Our model introduces the concept of a longitudinal, lecture-based climate change curriculum to assist in educating resident learners in evidence-based climate health knowledge, assist in preparing EPs to better treat climate-vulnerable patient populations, and share climate solutions.

## INTRODUCTION

The United Nations Intergovernmental Panel on Climate Change Sixth Assessment Report established that the average global surface temperature of the Earth between 2011–2020 has risen by 1.09 °C since the pre-industrial period (1850–1900) and correlated with increases in the atmospheric concentration of carbon dioxide.^1^ There is compelling evidence that global surface temperature rise has been primarily caused by human-driven greenhouse gas (GHG) emissions.^1^

Multiple studies have evaluated the impact of climate change on the medical field and vulnerable patient populations.^2^–^6^ The World Health Organization conservatively projected that by the 2030s an additional 250,000 deaths per year globally would be associated with climate change impacts.^7^ As climate-related health impacts intensify, emergency physicians (EP) will increasingly encounter patients whose conditions are influenced by environmental change. Recent literature has further looked at how the climate health changes will specifically affect emergency medicine (EM).^2^,^8^,^9^ Patients affected by heatwaves, flooding, weather disasters, cardiopulmonary illness exacerbated by poor air quality, and the expansion of vector-borne infectious diseases will seek care in the emergency department (ED).^2^ Therefore, EPs need to be educated on the impacts of climate change to be prepared to treat these patients.^2^,^6^,^8^,^9^ However, the literature is sparse on the implementation of a climate health curriculum in EM residency training.^10^,^11^

## OBJECTIVES

The purpose of our curricular intervention was to provide structured climate health education to EPs, EM residents, and medical students and to assess its effectiveness. Using a longitudinal lecture-based curriculum, we aimed to educate learners on climate health topics, discuss selected proposed climate solutions to ameliorate effects of climate change, and assess learners’ perceived understanding of the impacts of climate change on patient health in the ED. Physicians should understand both how to treat climate-vulnerable patients and the underlying global mechanisms by which their medical conditions are worsened. We aimed to prepare EPs for climate-related health emergencies and inspire them to support climate solutions.^12^

## CURRICULAR DESIGN

A longitudinal, climate-change educational curriculum was delivered in a four-part lecture series by an emergency attending physician or senior EM resident over three months during weekly EM resident didactic sessions in a three-year residency program. Our audience consisted of postgraduate year 1–3 EM residents, third- and fourth-year medical students who were on a one-month EM rotation, and academic emergency attending physicians. At this institution all participants receive an email describing the content of upcoming didactic sessions one week prior to the session; this also occurred during the months when the climate change curriculum was delivered. There was no additional formal recruitment of participants to the didactic sessions. While many participants likely attended more than one session, we did not track individual attendance.

The overall goal of the curriculum was to deliver educational content on climate-sensitive diseases that impact EM. While the scope of climate change and impact on health is broad, the focus was to introduce general climate-change concepts and tie those concepts to medical conditions encountered in the ED. The didactic presentations were modeled on evidence-based approaches to teaching learners with the goal of presenting scientific data in an objective manner, including discussing limitations of the available studies.

**Figure 1:**
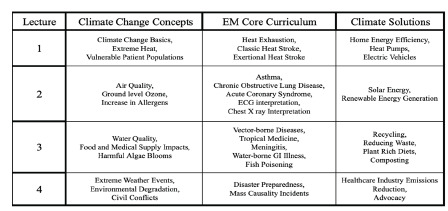
Overview and breakdown of topics covered in the four lectures of a climate health education curriculum. *EM*, emergency medicine; *ECG*, electrocardiogram; *GI*, gastrointestinal.

Didactic session #1 included an overview of foundational climate-change concepts and the impacts to the United States as outlined by the Fifth National Climate Assessment conducted by the National Oceanic and Atmospheric Administration.^13^ Additionally, the session introduced eight broad ways climate change is impacting human health.^5^ The initial lecture introduced the following climate topics: severe weather; air pollution; changes in disease vectors; increasing allergens; water quality; food and water supply impacts; environmental degradation; and extreme heat.^3^,^5^ Each subsequent didactic session was structured into three parts: deep dive into a climate change concept; teaching EM residency core curriculum (as per the guidelines of the Council of Residency Directors in Emergency Medicine)^15^ covering pathology exacerbated by climate change, and discussion of actionable climate change solutions to minimize GHG emissions (Figure 1).

To measure the effectiveness and learners’ perceived knowledge gained from the curriculum, survey data were collected at multiple points throughout the three-month period. Surveys were electronic, conducted anonymously via an email link or a QR code, completed voluntarily, and were accessible immediately after each didactic session was completed. Learners initially completed a pre-curriculum survey that was emailed out one week prior to the initial didactic session and available via QR code immediately prior to the first session. This survey assessed baseline knowledge of concepts to be covered in the curriculum. Post-curriculum surveys were distributed to the learners who attended each lecture immediately after the lecture and via email available for completion up to one week post lecture.

We initially intended to have one survey per lecture, but since lectures 1 and 2 were presented in the same didactic session, questions were combined into one survey that was sent out after lecture 2. These surveys included the same questions as the pre-curriculum survey, but only on topics specific to what had been taught thus far. For all surveys, learners were asked to rate responses on a 10-point Likert scale with 1 indicating “no confidence / no knowledge” and 10 indicating “very confident / very knowledgeable.” We assessed responses to the pre- and post-curriculum surveys using descriptive statistics and compared them using a Mann-Whitney U test to determine whether there was a change in median perceived knowledge or confidence level. A statistically significant change was defined as a *P* value ≤.05. Statistical analysis was performed using SAS v9.4 (SAS Institute Inc, Cary, NC).

The primary outcome used to assess effectiveness of the curriculum was a statistically significant improvement in the median perceived knowledge level of individual climate-change concepts before and after delivery of each section of the climate health curriculum. Secondary outcomes included assessment of learners’ belief in human-driven climate change, likelihood of performing individual action to combat climate change, and an open comments section.

## IMPACT AND EFFECTIVENESS

Nineteen learners completed the pre-curriculum survey. The three post-lecture surveys had a wide range of respondents from 7–27, which limited our analysis (Table 1). Overall, the majority of the respondents were EM residents (72.9%). Other respondents included medical students (20.3%) and attending physicians (6.8%). Table 1 shows the breakdown of respondents for each survey.

**Figure 2:**
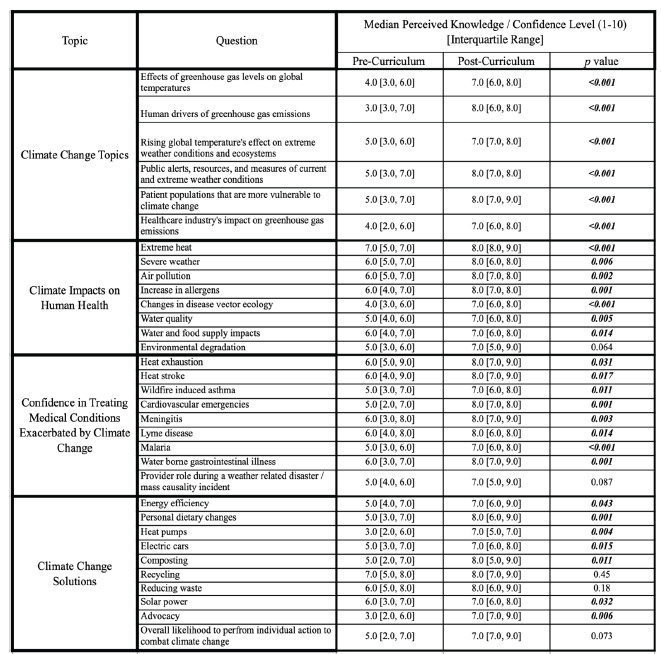
Comparison of participants’ median responses on a 10-point Likert scale between pre- and post-curriculum surveys in which they assessed a climate-related health curriculum.

In the pre-curriculum survey, 73.7% of respondents reported that they had never attended a lecture on climate change and its effect on human health. Climate change is not generally part of core medical school or graduate medical education curriculum.^11^,^14^ Therefore, the pre-curriculum survey provided valuable feedback demonstrating that many learners are not formally educated on this topic. When assessing individual topics that were presented, learners reported improved perceived knowledge or confidence in 87.5% of concepts (28/32 concepts with *P* < .05) that were assessed during the climate health educational curriculum (Figure 2). When assessing the four core areas of the curriculum individually—1) climate change topics, 2) climate impacts on human health, 3) confidence in treating medical conditions exacerbated by climate change, and 4) climate change solutions—the survey demonstrated improvement perceived knowledge in all four areas.

Specifically, when evaluating perceived knowledge of the six core climate-change topics, every area showed statistically significant improvement in perceived knowledge (*P* < .05), demonstrating in our small cohort of learners that an introduction to climate change in a residency-based educational curriculum could have a large impact on climate change knowledge for future physicians. In the additional core topic areas, there was a reported increase in perceived knowledge level in 7 of 8 ways that climate change impacts human health (Figure 2). Learners also reported statistically significant improved confidence in treating 8 of 9 medical conditions exacerbated by climate change (Figure 2). Lastly, learners demonstrated statistically significant improved perceived knowledge of 8 of 10 climate change solutions (Figure 2). In the last question on the survey regarding ability to perform climate-change solutions, learners reported that they were more likely to perform individual climate solutions in the following six months after the conclusion of the climate health educational curriculum; however, this difference was not statistically significant and was limited by the low number of survey respondents to the final survey (5.0 vs 7.0, *P* = .073).

In the open comments sections, learners stated that they had gained knowledge on the compounding effect of climate change on health, impacts of food waste, better management of gastrointestinal illness and meningitis, and various environmental impacts on different pathologies. Encouragingly, even before completion of the curriculum, 48.1% of respondents stated that they had implemented climate solutions including carpooling, composting, wasting less food, changing diet, conserving energy, and changing home energy suppliers to renewable resources.

A limitation of this pilot curricular study is the small study size limited to one academic center focused on EM resident education. The varied number of survey responses, from 7–27 respondents, limits the power of our statistical analysis and any definitive conclusions as to the curriculum’s effectiveness. It is suspected that the low response rate to the final survey was due to the lecture being scheduled as the last of one of the didactic sessions, which extended into lunch break. Despite follow-up emails, the survey response rate remained low. Timing of the lectures within the didactic session is something to consider for future curriculum building. Additionally, since learners were self-reporting a perceived knowledge benefit and we did not perform a knowledge assessment, we are unable to comment on long-term retention. Finally, responses were analyzed as a whole and not broken down by level of education due to the small sample size. One would expect that a change in perceived knowledge level at baseline would differ between a medical student and an attending physician.

Regardless of these limitations, the goal of the project was to introduce a curriculum to EM residents that had not been presented to them previously and was likely an area of reduced knowledge across residency programs, based on literature review.^11^,^14^ This project demonstrates that a simple, lecture-based curriculum introducing climate-related health concepts can raise awareness of general climate issues, including how climate change is likely to impact ED patients. Future studies should assess knowledge retention and integration over the span of EM residency and could improve on our design to align with published, public- health core climate and health competencies such as those proposed by the Global Consortium on Climate and Health Education, as our curricular design was not based on published climate health competencies.^16^

## CONCLUSION

It is pivotal that current and future emergency physicians be aware of climate change and its effects on human health and be trained to treat climate-vulnerable patients, as well as to understand how to become advocates for climate change solutions. Our longitudinal curriculum improved learners’ overall perceived understanding and knowledge of climate change, its impact on patient health, and treating medical conditions exacerbated by climate change. After attending the course, learners reported that they were more likely to implement climate change solutions into their personal lives or had already implemented them. Although there are limitations to survey studies, we are encouraged by the positive results of our curriculum design and analysis. This intervention demonstrates one way to integrate climate health knowledge into the core EM residency curriculum. We encourage EM residencies to use this model and build on it to disseminate evidence-based, climate health knowledge to prepare physicians to treat climate-vulnerable patient populations and share climate solutions.

## Figures and Tables

**Table 1:** Total number of respondents to survey regarding a climate-change curriculum by level of education.

Breakdown of Survey Respondents				
Survey	Students	Residents	Attending Physicians	Total
Pre-Curriculum	5	13	1	19
Post Lectures 1&2	6	14	1	21
Post Lecture 3	4	21	2	27
Post Lecture 4	0	6	1	7
